# Statistical optimization for cadmium removal using *Ulva fasciata* biomass: Characterization, immobilization and application for almost-complete cadmium removal from aqueous solutions

**DOI:** 10.1038/s41598-018-30855-2

**Published:** 2018-08-20

**Authors:** Noura El-Ahmady El-Naggar, Ragaa A. Hamouda, Ibrahim E. Mousa, Marwa S. Abdel-Hamid, Nashwa H. Rabei

**Affiliations:** 1 Department of Bioprocess Development, Genetic Engineering and Biotechnology Research Institute, City of Scientific Research and Technological Applications, Alexandria, Egypt; 2 Department of Microbial Biotechnology, Genetic Engineering and Biotechnology Research Institute, University of Sadat City, 22857, Menoufyia Governorate, Egypt; 3 Department of Environmental Biotechnology, Genetic Engineering and Biotechnology Research Institute (GEBRI), University of Sadat City, 22857 Menoufyia Governorate, Egypt

## Abstract

Cadmium is a global heavy metal pollutant. Marine green algae were used as efficient, low cost and eco-friendly biosorbent for cadmium ions removal from aqueous solutions. Plackett-Burman design was applied to determine the most significant factors for maximum cadmium removal from aqueous solutions using dry *Ulva fasciata* biomass. The most significant factors affecting cadmium removal process were further optimized by the face centered central composite design. The results indicated that 4 g of dry *Ulva fasciata* biomass was found to successfully remove 99.96% of cadmium from aqueous solution under the conditions of 200 mg/L of initial cadmium concentration at pH 5, 25 °C for 60 min of contact time with static condition. Dry *Ulva fasciata* biomass samples before and after cadmium biosorption were analyzed using SEM, EDS and FTIR. Furthermore, the immobilized biomass in sodium alginate-beads removed 99.98% of cadmium from aqueous solution at an initial concentration of 200 mg/L after 4 h which is significantly higher than that for control using sodium alginate beads without incorporation of the algal biomass (98.19%). Dry biomass of *Ulva fasciata* was proven to be cost-effective and efficient to eliminate heavy metals especially cadmium from aquatic effluents and the process is feasible, reliable and eco-friendly.

## Introduction

Few metals such as iron, copper, manganese, cobalt, zinc are essential for growth and metabolism in living organisms and are considered as micronutrient^1,2^. However, higher concentrations of these metals are toxic and have adverse effects on the environment. Unlike many other contaminants, the heavy metals are known to be powerful inhibitors to natural biodegradation processes of organic contaminants and cannot be easily removed from the environment^3^. These metals cannot be chemically or biologically degraded and are ultimately indestructible. Heavy metals are major pollutants of freshwater reservoirs and represent an important environmental problem because of their toxic, non-biodegradable and persistent nature^4,5^. Heavy metals ions are easily absorbed by fishes and vegetables because of their high solubility in the aquatic environments. Thus, they may accumulate in the human body tissues through the food chain^4^. Heavy metal contamination has raised serious concerns because it can cause harmful health effects on humans, especially reproductive abnormalities, fetal death, and neurological and behavioral disorders^6^.

In recent years, many industries (such as energy and fuel producing, fertilizers, mining, surface finishing, iron and steel, pesticides, electroplating, electrolysis, leather photography and atomic energy) discharge their wastes containing different heavy metals directly or indirectly into the environment, causing serious environmental pollution and even threatening human life^7,8^. It is well known that heavy metals discharged into the environment by industrial wastewaters are a major environmental problem owing to their toxicity to humans and to other living species^9^. Reducing the toxicity of heavy metals is a major challenge for all developing countries. Because of the high cost, industries avoid industrial wastes management which increases the discharges of large amounts of potential hazard heavy metals to the environment.

Cadmium (Cd) is an extremely toxic industrial and environmental pollutant heavy metal. It cannot be biodegraded and accumulated throughout the food chain from uptake by plants in the polluted soils and can be very dangerous to human health. Maximum concentration allowed in drinking water (ppm) for cadmium in US EPA (0.005) Singapore, 0.003 and in China, 0.01. Toxicity caused by cadmium has been reported by many authors^10,11^. Ingestion of any significant amount of cadmium causes immediate poisoning and damage to the liver. It causes kidney disease through renal tubule dysfunction^12^. Also, cadmium is reported to be a toxic metal for endocrine organ including pituitary^13^. Compounds containing cadmium act as a potent carcinogen^14^. Hotz *et al*.^15^ reported that there is sufficient evidence in humans for the carcinogenicity of cadmium and cadmium compounds. Chronic exposure to elevated levels of cadmium is known to cause renal dysfunction, bone degeneration, liver damage and blood damage^16^.

The conventional treatment techniques for removal of heavy metals contaminants from the environment or wastewater effluents include ion exchange, membrane filtration, chemical reduction and precipitation, adsorption on activated carbon, nanotechnology treatments, electrochemical removal and advanced oxidation processes^4,17^. Unfortunately, many of these conventional processes are limited by their significant disadvantages such as low selectivity, high coast, incomplete removal, high energy consumption or generate large amounts of toxic wastes^18^. So, the need for safe, cheaper and more efficient methods to eliminate heavy metals from polluted water has required research attention towards low cost alternative method to the commercially available methods^19^. Biochar and compost used as a sorbent for the remediation of soils contaminated with heavy metals^20,21^. Zeng *et al*.^22^ and Wu *et al*.^23^ studied the efficiency of biochar and compost as effective *in situ* remediation materials for heavy metals in contaminated soils. These materials decreased the available heavy metals concentrations in the soil and soil pore water. Composted biochar had the greater capacity for reducing the bioavailability and mobility of heavy metals. Biosorption of heavy metals by natural materials provide potential alternative and promising technology to overcome the disadvantages of conventional methods to remove heavy metal ions from aqueous solutions and wastewater. A variety of natural biosorbents have been used to separate heavy metals from water and wastewaters such as peat, olive core, grape shoot and several marine macro algae such as biomass of green algae^24^.

Biosorption process refers to the use of living organisms, primarily microorganisms (fungi, bacteria, algae, and yeasts) as biosorbents to degrade or detoxify the environmental contaminants hazardous to human health and/or the environment (like heavy metals in polluted water and lands) under controlled conditions into less toxic forms with levels below concentration limits established by regulatory authorities^25^. Several studies have shown that the main advantages of heavy metals removal from wastewater by microorganisms over conventional treatment methods include lower cost, better performance, high efficiency, minimization of chemical and/or biological sludge, regeneration of biosorbent and the possibility of metal recovery^26^. Microorganisms rely on different mechanisms for detoxification such as biosorption, biomineralization and biotransformation, which can be exploited for biological treatment (bioremediation) either *in situ* or *ex situ*^27,28^.

Marine green algae are among the most promising organisms that have a great potential for heavy metal removal due to their large surface area and rapid rate of metal accumulation from aqueous solutions^29^. These algae are efficient and cheap biosorbents as the nutrition requirements by algae are low and don’t produce toxic substances^30^ and possess a high metal binding capacity^31^. The mechanism of metal binding capacity of marine green algae is mainly based on physical adsorption and/or chemical adsorption by covalent binding between negative charge of the cell surface and the various functional groups of polysaccharides, proteins and lipids on the cell wall surface^32^. Tuzun *et al*.^33^ recorded that the metal biosorption depends on the biomacromolecules on the algal surface that contains a variety of functional groups (such as, amino, carboxyl, thiol, sulfhydryl and phosphate groups), these groups serve as adsorption sites. Ghouneim *et al*.^34^ concluded that the dead biomass of green alga *Ulva lactuca* could be used as efficient, low cost, and environmentally friendly biosorbent material for the removal of cadmium ions from aqueous solutions. *Ulva fasciata* is a widespread macro algae occurring at all levels of the intertidal zone, in calm and protected harbors and used in the current study for the removal of cadmium ions from aqueous solutions.

The main objectives of the present study is to investigate the biosorption characteristics of the biomass of marine green alga, *Ulva fasciata*, for the removal of cadmium ions from aqueous solutions, the statistical optimization for cadmium removal, biomass characterization before and after cadmium biosorption using SEM, FTIR and EDS, in addition to immobilization of *Ulva fasciata* in alginate beads and its application in cadmium removal.

## Results and Discussion

The biosorption process is a complex system and its efficiency is significantly influenced by several variables including the biomass as adsorbent material that capable of the adsorption of the heavy metal; the availability and concentration of the heavy metal; the physicochemical factors like temperature, pH, dissolved oxygen and contact time. The effect of these factors on the biosorption of cadmium has been studied. Improving the efficiency of heavy metals biosorption using the classical method is tedious, laborious, time-consuming and expensive for a large number of factors. These limitations of the classical method can be eliminated by optimization using Plackett-Burman and face-centered central composite designs which reduces the number of the experiments, simplifies improvement by studying the effect of individual and mutual interactions between the variables on the response.

### Evaluation of variables affecting cadmium removal by *Ulva fasciata* using Plackett-Burman design

Plackett-Burman design was applied to determine the most significant factors for maximum cadmium removal from aqueous solutions using *Ulva fasciata* dry biomass. The alga was dried in an oven at 70 °C for 72 hrs and then milled with a blender, sieved to get particle with the size pass through a laboratory test sieve (Endecotts Ltd., London, England) with mesh size of 125 µm (Supplementary Fig. S1). The Plackett-Burman design matrix selected for the screening of significant variables along with the corresponding cadmium removal percentage is shown in Table 1. The experiment was conducted in 12 runs to study the effect of the selected six variables on the cadmium removal percentage. Plackett-Burman experiments showed a markedly wide variation (90.73 to 99.4%) in cadmium removal percentages; this variation reflected the importance of the optimization process to attain maximum cadmium removal. The relationship between cadmium removal percentage and the independent variables is determined by multiple-regression statistical analysis and the analysis of variance (ANOVA) of the experimental design was calculated and summarized in Table 2. Table 2 and Supplementary Fig. S2 show the estimated effect of each variable on cadmium removal percentage. The large effect, either negative or positive, indicates that the variable has a large impact on cadmium removal, whereas the near zero effect means that the variable has little or no effect. The contributions percentages for each variable are given in Table 2.

**Table 1:** Twelve-trials Plackett–Burman experimental design for evaluation of independent variables with coded and actual levels along with the observed and predicted values of cadmium removal by *Ulva fasciata*.

Run no.	Coded and actual levels of independent variables												Cadmium removal (%)		Residuals
	Contact time (min.)		Cadmium conc. (mg/L)		pH		Temperature (°C)		Biomass (g/L)		Agitation -Static		Actual value	Predicted value	
1	−1	60	−1	25	−1	4	1	50	1	4	1	Agitation	92.3	92.789	−0.489
2	−1	60	−1	25	1	7	1	50	1	4	−1	Static	94.66	93.763	0.898
3	−1	60	1	200	1	7	1	50	−1	1	1	Agitation	99.14	99.104	0.036
4	1	180	−1	25	1	7	1	50	−1	1	1	Agitation	91.5	91.944	−0.444
5	1	180	−1	25	−1	4	−1	25	1	4	1	Agitation	91.65	91.526	0.124
6	−1	60	1	200	−1	4	−1	25	−1	1	1	Agitation	97.9	97.431	0.469
7	1	180	1	200	−1	4	1	50	−1	1	−1	Static	97.5	97.473	0.028
8	1	180	1	200	1	7	−1	25	1	4	1	Agitation	99.4	99.096	0.304
9	−1	60	−1	25	−1	4	−1	25	−1	1	−1	Static	90.73	90.834	−0.104
10	1	180	−1	25	1	7	−1	25	−1	1	−1	Static	91.26	91.244	0.016
11	1	180	1	200	−1	4	1	50	1	4	−1	Static	98.7	98.728	−0.027
12	−1	60	1	200	1	7	−1	25	1	4	−1	Static	98.85	99.659	−0.809

The −1 sign correspond to the minimum value and the +1 sign correspond to the maximum value of the input parameter range.

**Table 2:** Regression statistics and analysis of variance (ANOVA) for the experimental results of Plackett-Burman design used for cadmium removal by *Ulva fasciata*.

Source	*df*	Coefficient	Effect	Contribution %	*t -*Stat	*P*-value	Confidence Level (%)
Model	6	95.2992			493.355	0.00024	99.976
Contact time (A)	1	−0.2975	−0.595	5.879	−1.540	0.184	81.60
Cadmium conc. (B)	1	3.2825	6.565	64.872	16.993	0.00001	100.00
pH (C)	1	0.5025	1.005	9.931	2.601	0.048	95.20
Temperature (D)	1	0.3342	0.6684	6.605	1.730	0.144	85.60
Biomass (E)	1	0.6275	1.255	12.401	3.249	0.023	97.70
Agitation –Static (F)	1	0.0158	0.0316	0.312	0.082	0.938	6.20
	***df***	***SS***	***MS***	***F***			
Regression	6	139.458	23.243	51.910			
Residual Error	5	2.239	0.448				
Total	11	141.697					
R	0.9921	R^2^ (adj)	0.9652				
R^2^	0.9842	R^2^ (pred)	0.9090				

*Significant values, *df*: Degree of freedom, *F*: Fishers’s function, *P*: Level of significance, PRESS 12.8953.

The results revealed that contact time (A), temperature (D) and agitation/static (F) are three insignificant variables with lower effects (−0.595, 0.6684 and 0.0316; respectively) and lower percent of contribution (5.879, 6.605 and 0.312; respectively). The ANOVA of the Plackett-Burman design demonstrated that the model was highly significant as was evident from the Fisher’s *F*-test (51.910) with a very low probability value [*P-* value = 0.00024] and the *t*-Stat of 493.355. Also factors evidencing *P*-values less than 0.05 were considered to have significant effects on cadmium removal.

The results showed that cadmium concentration (B) having a probability value of 0.00001 was the most significant variable affecting cadmium removal by *Ulva fasciata* followed by biomass (E) and pH (C) with probability values of 0.023 and 0.048; respectively, the lower probability values indicate the more significant factors affecting cadmium removal.

The coefficient of each factor represents the effect extent of this factor on cadmium removal. Moreover, it was clear that the cadmium concentration, pH, temperature, biomass and agitation/static had positive effects on cadmium removal (the coefficient values are 3.282, 0.502, 0.334, 0.627 and 0.015; respectively). Among the non-significant variables, the contact time has a negative effect on cadmium removal (the coefficient value is −0.297), which means that the increase in pH, temperature, biomass and agitation/static levels and decrease in contact time level could exert a positive effect on cadmium removal.

The goodness of fit of the model was checked by the determination coefficient (R^2^). In our study, the value of the determination coefficient (R^2^ = 0.9842) indicated that 98.42% of the variability in the response could be explained by the model and only 1.58% of the total variations are not explained by the model. In addition, the value of the adjusted determination coefficient (Adj. R^2^ = 0.9652) was also very high and ensure a high significance of the model (Table 2). The higher value of the Adj. R^2^ refers to more accuracy of the relationships between the studied experimental factors and cadmium removal. At the same time, the predicted-R^2^ value of 0.9090 for the cadmium removal by *Ulva fasciata* was in a reasonable agreement with the Adjusted-R^2^ of 0.9652 which indicated that the model is good. The R^2^-prediction indicates how well the model expects responses for new experiments, and therefore our results revealed that the cadmium removal is predictable with 90.90% accuracy.

**Figure 1:**
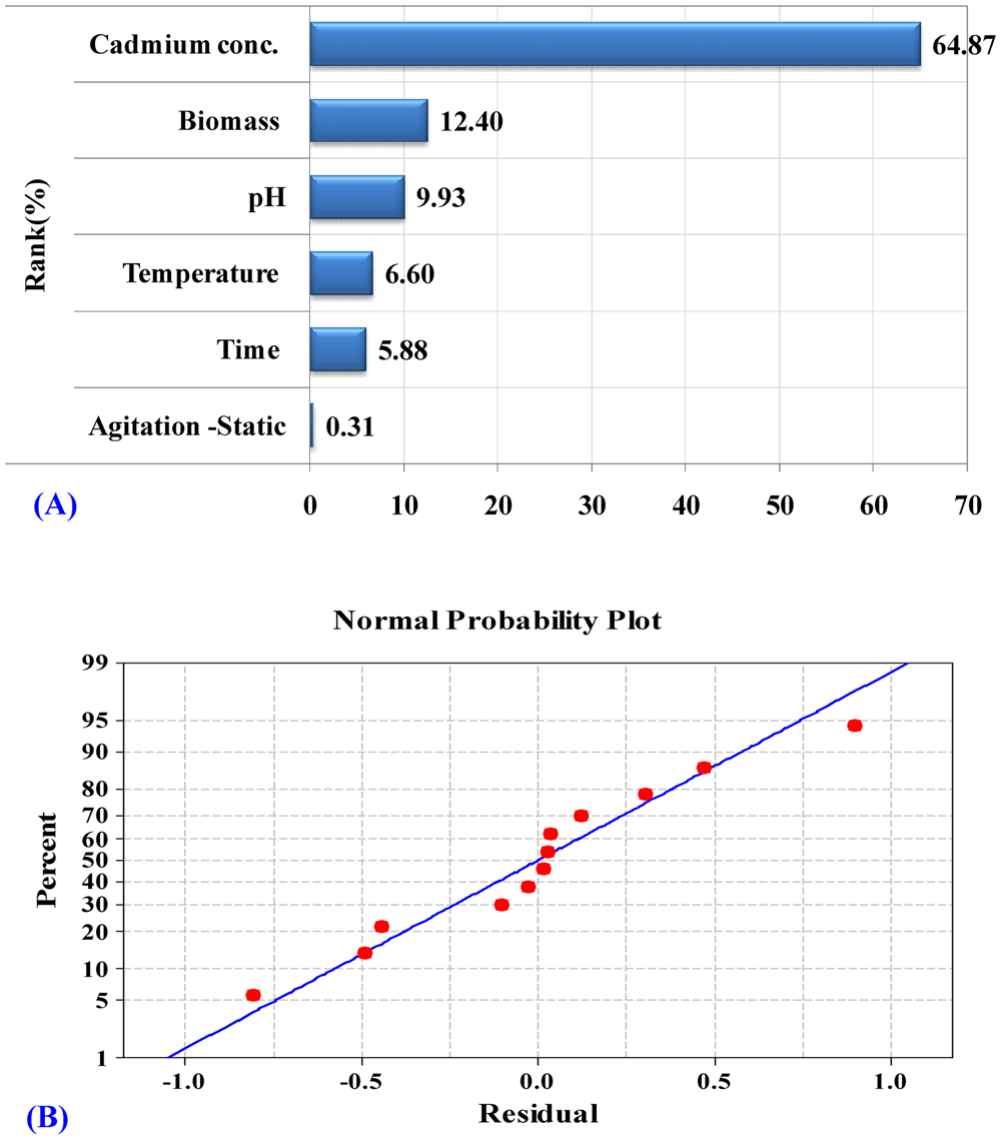
(**A**) Pareto chart illustrates the order and significance of the variables affecting cadmium removal by *Ulva fasciata* using Plackett-Burman design; Ranks (%) values ranging from 0.31 to 64.87); (**B**) The normal probability plot of the residuals for cadmium removal by *Ulva fasciata* determined by the first-order polynomial equation.

Pareto chart (Fig. 1A) showed that cadmium concentration (B) was the most significant variable affecting cadmium removal (64.87%) followed by biomass (E), pH (C), temperature (D), contact time (A), then agitation/static (F).

### The normal probability plot (NPP) of the residuals

NPP of the residuals is an important graphical technique to check the adequacy of the model^35^. Figure 1B shows the NPP of the experimental results. By plotting the residuals versus the expected values of the model, the residuals show the points on such a plot should fall close to the diagonal reference line. The deviations from this straight line suggest the residuals departures from normality. In this study, the normal probability plot of the residuals shows the residuals from the fitted model are close to the diagonal line and appear to be normally distributed. This indicates that the model was well fitted with the experimental results.

A first-order polynomial equation describing the relationship between the independent variables and the cadmium removal percentage was derived. By neglecting the insignificant variables the following equation was obtained in terms of coded variables:

1 $${\rm{Cadmium}}\,{\rm{removal}}\,( \% )=95.299+3.282\,{\rm{B}}+0.502{\rm{C}}+0.627\,{\rm{E}}$$

where B, C, and E are cadmium concentration, pH and biomass concentration; respectively.

On the basis of *P-*values (Table 2), cadmium concentration, pH and biomass concentration were chosen for further optimization using face centered central composite design (FCCD), since these variables had the most significant effects on cadmium removal.

In a confirmatory experiment, to evaluate the accuracy of Plackett-Burman, the conditions which expected to be optimum for maximum cadmium removal by *Ulva fasciata* biomass from aqueous solutions were contact time of 60 min, initial cadmium concentration of 200 mg/L, pH 7, temperature 25 °C, *Ulva fasciata* biomass of 4 g/L at static condition. Under these conditions, the maximum removal percentage of cadmium was 99.2% which is higher than the removal percentage of cadmium obtained before applying Plackett-Burman (50.3%) by 1.97 times.

### Statistical optimization of cadmium removal by *Ulva fasciata* biomass using face centered central composite design (FCCD)

Results of Plackett-Burman design revealed that cadmium concentration (X_1_), pH (X_2_) and biomass (X_3_) were the most significant factors affecting cadmium removal and thus were selected to further optimize using face centered central composite design. Table 3 shows the three factors and their three levels (coded and actual levels) used in the design matrix. Contact time which exerted a negative effect on cadmium removal and other insignificant variables were maintained in all trials at their low levels of Placket-Burman design for further optimization by face centered central composite design.

**Table 3:** Face centered central composite design representing the experimental cadmium removal percentages by *Ulva fasciata* as influenced by cadmium concentration (X_1_), pH (X_2_) and biomass (X_3_) along with the predicted cadmium removal percentages and residuals and the actual factors levels corresponding to coded factors levels.

Std	Run	Type	Variables			Cadmium removal (%)			Residuals
			X_1_	X_2_	X_3_	Experimental		Predicted	
4	1	Factorial	1	1	−1	93.81		93.37	0.44
18	2	Center	0	0	0	99.86		98.35	1.51
10	3	Axial	1	0	0	93.57		94.81	−1.24
13	4	Axial	0	0	−1	99.95		100.64	−0.69
11	5	Axial	0	−1	0	99.96		100.81	−0.85
20	6	Center	0	0	0	99.95		98.35	1.60
19	7	Center	0	0	0	97.90		98.35	−0.45
7	8	Factorial	−1	1	1	89.22		88.90	0.32
3	9	Factorial	−1	1	−1	99.83		99.94	−0.11
15	10	Center	0	0	0	97.99		98.35	−0.36
2	11	Factorial	1	−1	−1	98.56		98.46	0.10
1	12	Factorial	−1	−1	−1	98.8		98.55	0.25
9	13	Axial	−1	0	0	90.71		91.15	−0.44
12	14	Axial	0	1	0	99.69		100.52	−0.83
6	15	Factorial	1	−1	1	98.81		98.29	0.52
17	16	Center	0	0	0	99.86		98.35	1.51
16	17	Center	0	0	0	97.90		98.35	−0.45
8	18	Factorial	1	1	1	96.50		96.33	0.17
5	19	Factorial	−1	−1	1	84.36		84.38	−0.02
14	20	Axial	0	0	1	94.04		95.03	−0.99
**Variable**			**Variable code**		**Coded and actual levels**				
					**−1**	**0**	**1**		
Cadmium conc. (mg/L)			X_1_		100	200	300		
pH			X_2_		5	7	9		
Biomass concentration (g/L)			X_3_		2	4	6		

Experimental and predicted cadmium removal percentages for the twenty trials of the employed design matrix are presented in Table 3. The results show considerable variation in the percentages of cadmium removal depending on the three independent variables variations. Based on the experimental data obtained; cadmium removal percentage ranged from 84.36 to 99.96%. The highest percentage of cadmium removal was obtained in the run 5 with a value of 99.96%, where cadmium concentration is 200 mg/L, biomass is 4 g/L and pH is 5, while the minimum cadmium removal percentage was observed in the run number 19 with a value of 84.36% where cadmium concentration is 100 mg/L, pH is 5 and biomass is 6 g/L were used.

### Multiple regression analysis and ANOVA

The FCCD results were analyzed using multiple regression analysis which is represented in Table 4. The present regression model has R^2^ value = 0.9637 which indicated that 96.37% of cadmium removal variations was attributed to the independent variables and only 3.63% of the total variations cannot be explained by the model. A regression model with an R^2^-value above 0.9 is considered to have a very high correlation^36^.

**Table 4:** Regression statistics of FCCD, regression coefficients and analysis of variance (ANOVA) for FCCD results used for cadmium removal percentage by *Ulva fasciata*.

Source	*F-*value	*P-*value *P*rob > *F*	Coefficient estimate
Model	29.497	<0.0001*	98.351
X_1_ - (Cadmium conc., mg/L)	25.975	0.0005*	1.833
X_2_ - (pH)	0.160	0.6973	−0.144
X_3_ - (Biomass conc., g/L)	60.698	<0.0001*	−2.802
X_1_ X_2_	16.206	0.0024*	−1.619
X_1_ X_3_	75.710	<0.0001*	3.499
X_2_ X_3_	3.799	0.0799	0.784
X_1_^2^	61.339	<0.0001*	−5.371
X_2_^2^	11.380	0.0071*	2.314
X_3_^2^	0.567	0.4689	−0.516
Std. Dev.	1.137	R-Squared	0.9637
Mean	96.564	Adj R-Squared	0.9310
C.V.%	1.178	Pred R-Squared	0.8776
PRESS	43.628	Adeq Precision	20.4227

*Significant values, *F*: Fishers’s function, *P*: Level of significance.

Lack of Fit: 1.241; lack of fit *P*-value = 0.4094.

In addition, the value of the adjusted determination coefficient (Adj R^2^ = 0.9310) was also very high to confirm the high significance of the model (Table 4). The predicted R^2^ value of 0.8776 was in a reasonable agreement with the adjusted R^2^ value. This indicated a good match between all of the experimental values and their predicted values. At the same time, a relatively lower value of the coefficient of variation percentage (C.V. = 1.178%) indicates a better precision and reliability of the experiments carried out^37^. The adequate precision value of the present model was 20.422 and this also suggests that the model can be used to navigate the design space. The predicted residual sum of squares (PRESS) is a measure of the suitability of the model for each point in the design. A regression model with a small PRESS value is the best model that fits the data points. Our PRESS value is 43.628. The mean and standard deviation value of the model are 96.564 and 1.137; respectively (Table 4). The positive coefficients indicate the increase in cadmium removal, while negative coefficients indicate the decrease in cadmium removal.

The ANOVA of the quadratic regression model demonstrates that the model is highly significant as evident from the Fisher’s *F* test (*F* value = 29.497) with a very low probability value [*P-*value Prob > *F* less than 0.0001] (Table 4). If the Prob > *F* values are very small (less than 0.05) indicates that the model terms are significant.

The significance of each coefficient was determined by *P*-values as listed in Table 4. The interpretation of the data was based on signs (negative or positive effect on cadmium removal) and statistical significance of the coefficients (*P* < 0.05). The effects of the interactions between two variables can be explained as a positive sign (+) of the coefficients means a synergistic effect on the cadmium removal, whereas a negative sign means an antagonistic effect. It can be seen from the degree of significance that the linear coefficients of X_1_, X_3_, the interaction between X_1_ X_2_ and X_1_X_3_, the quadratic effect of X_1_ and X_2_ are significant. Furthermore, the probability values of the coefficients suggest that among the three variables studied, the interaction between X_1_ and X_3_ had a very significant effect on cadmium removal by *Ulva fasciata* with a probability value of <0.0001, indicating that <9.99% of the model affected by cadmium concentration (X_1_) and biomass concentration (X_3_). On the other hand, the interaction between X_1_ and X_2_ (cadmium concentration and pH) had a significant effect with a probability value of 0.0024. The linear coefficients of X_2_, the interaction between X_2_ and X_3_, the quadratic effect of X_3_ are not significant model terms that not contribute to the response (cadmium removal percentage).

Supplementary Table S1 shows the fit summary results which contributed to select the highest order polynomial model where the lack of fit test is insignificant and the terms are significant, also the model summary statistics focus on the model that has lower standard deviation, higher adjusted and predicted R-squared. The fit summary results showed that, the quadratic model is a highly significant and adequate model fitting the FCCD of cadmium removal by *Ulva fasciata* with a very low probability value (*P-*value < 0.0001), also lack of fit *F*-value 1.241 (the lack of fit is not significant, *P-*value = 0.4094). Summary statistics of the quadratic model (Supplementary Table S1) showed the smallest standard deviation of 1.1373 and the largest adjusted and predicted R-squared of 0.9310 and 0.8776; respectively.

The coefficients of the regression equation were calculated and the data (Table 4) was fitted to a second-order polynomial equation. The cadmium removal percentage (Y) by *Ulva fasciata* can be expressed in terms of the following regression equation:

2 $$\begin{array}{c}{\rm{Y}}=+\,98.351+1.833\,{{\rm{X}}}_{1}-\,0.144\,{{\rm{X}}}_{2}-2.802\,{{\rm{X}}}_{3}-\,1.619\,{{\rm{X}}}_{1}{{\rm{X}}}_{2}+\,3.499\,{{\rm{X}}}_{1}{{\rm{X}}}_{3}\\ \,\,+\,0.784\,{{\rm{X}}}_{2}{{\rm{X}}}_{3}-5.371\,{{{\rm{X}}}_{1}}^{2}+2.314\,{{{\rm{X}}}_{2}}^{2}-0.516\,{{{\rm{X}}}_{3}}^{2}\end{array}$$

where Y is the predicted value of cadmium removal percentage, X_1_, X_2_ and X_3_ are the coded levels of cadmium concentration, pH and biomass concentration.

### Contour and three dimensional (3D) plots

The graphical representation (3D plot and its corresponding contour plot) provides a method for visualizing the relationship between the response and the interactions among test variables in order to determine the optimum conditions for cadmium removal. 3D plots for the pair-wise combinations of the three variables (X_1_ X_2_, X_1_ X_3_ and X_2_ X_3_) were generated by plotting the response (cadmium removal percentage) on Z-axis against two independent variables while keeping the other variable constant at its center point (zero level).

**Figure 2:**
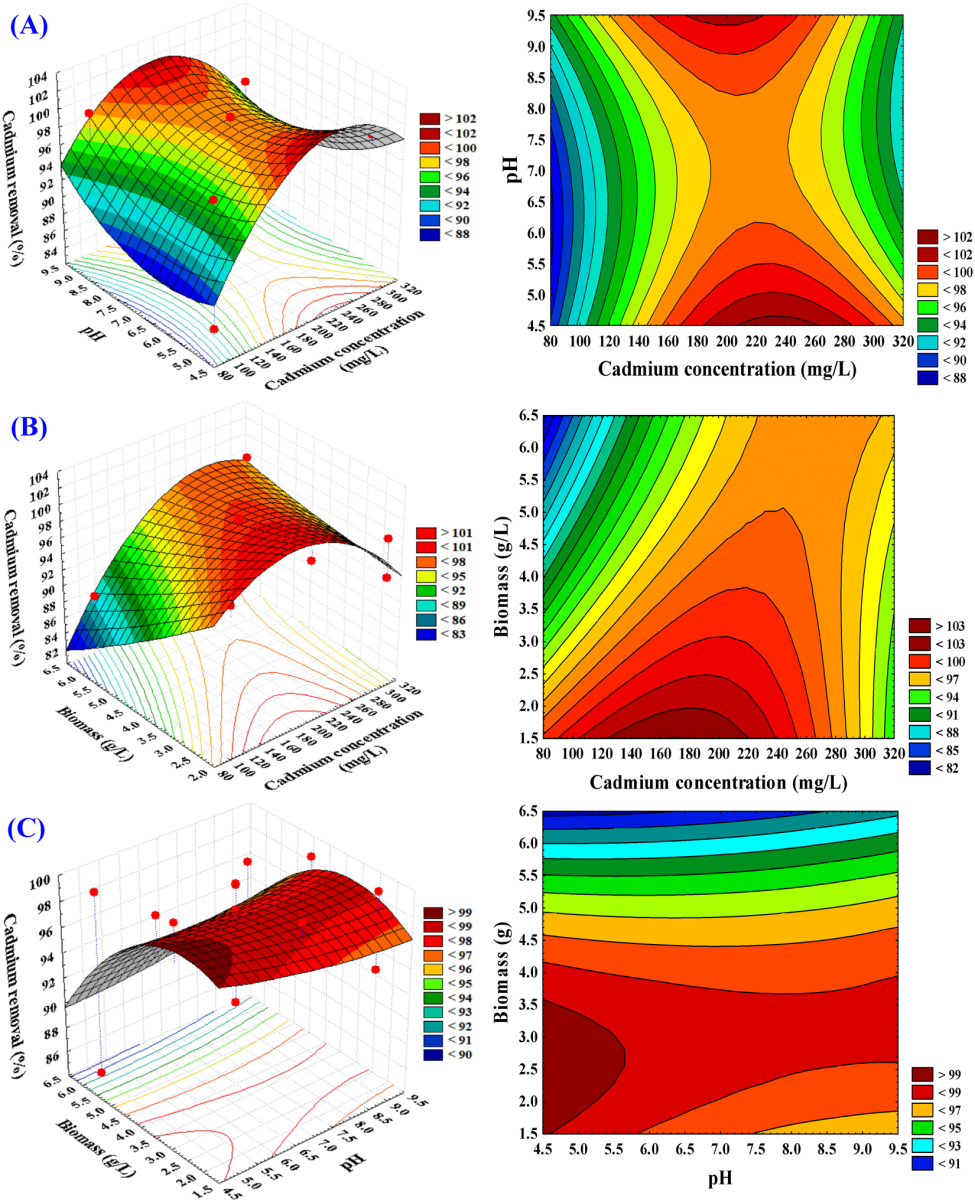
3D response surface and contour plots of the effects of cadmium concentration (X_1_), pH (X_2_) and biomass (X_3_) and their mutual effect on cadmium removal by *Ulva fasciata*.

The 3D plot and its corresponding contour plot (Fig. 2A), showing the effects of cadmium concentration (X_1_) and pH (X_2_) on cadmium removal percentage, while biomass (X_3_) was kept at its zero level (4 g/L). Figure 2A shows that lower and higher levels of cadmium concentration (X_1_) support a relatively low percentage of cadmium removal. On the other hand, the maximum percentage of cadmium removal clearly situated close to the central point of cadmium concentration. Lower and central levels of pH (X_2_) support the maximum percentage of cadmium removal while further increase in the level of pH resulted in a gradual decrease in the percentage of cadmium removal. By solving the Equation (2) and analysis of Fig. 2A, the maximum predicted cadmium removal of 100.81% was obtained at the optimum predicted levels of cadmium concentration and pH of 200 mg/L and 5; respectively at biomass concentration of 4 g/L.

The biosorption of different metal ions increased with increasing metal ion concentration and reached the maximum. However, the further increase in metal concentration leads to a slight increase in the removal percentage. This can be attributed to the fact that all active sites on the surface area of algal biomass on which the competitive adsorption of metal ions occurred were free at the beginning, resulting in high metal adsorption. Thereafter, with increasing metal concentration, the removal of metal was decreased due to a few binding sites that were available on the surface of algae^38^. Cadmium concentration is one of the most important factors that affect the cadmium removal, as initial metal ion concentration strongly influences the metal uptake in adsorption of metal ions^39^. Increasing metal ion concentration increased adsorption capacity for the adsorbent, could be attributed to increase the rate of mass transfer due to increased concentration of driving force^40^. The maximum metal adsorption may be due to the higher active site for algal powder which enhances metal ion uptakes^41,42^. In other studies, it was reported that each algal species has a preference for cadmium removal^43^.

Since, the pH is related to the net charge on the cell surface of the biosorbent, which determines the range of the cellular sites occupied by protons and other ions, the competition between heavy metals and H^+^ for same cellular binding sites can lead to a decrease or increase in sorption and toxicity of heavy metals, highly dependent on pH of the solution^44^. Moreover, the pH value affects the solubility of the metal ions in solution^45^. High alkaline pH value causes a decrease in the metals solubility, which causes a low absorption rate^46^. Moreover, the dependence of metal ions adsorption by biomass on pH can be justified by the association–dissociation of certain functional groups present on the surface of the biomass, such as carboxyl as well as hydroxyl groups. Most carboxyl groups are not dissociated at low pH and cannot bind the metal ions in solution^47^. At pH 5–6, the electrostatic repulsions between the surface of adsorbents and the positively charged metal ions are depressed, which increase the percentage of metal removal^48–50^. The decrease in the adsorption capacity at higher pH values could be related to the repulsion between the negative charge of anionic species in solution and negative surface charge of the sorbents^51,52^.

Our results revealed that the maximum cadmium removal was recorded at pH 5. The decreased biosorption rate of the cadmium at the cell surface at alkaline pH value may be because of the increased ability of protons to compete with cadmium ions for the binding sites in the biomass. Liu *et al*.^53^ reported that the maximum biosorption of cadmium ions from aqueous solutions by chemically pretreated biomass of brown alga *Laminaria japonica* was at pH value in the range of 4.3–6.5. Ghouneim *et al*.^34^ indicated that pH 5.5, 0.1 g of *Ulva lactuca* was enough to remove 99.2% of 10 mg/L cadmium at 30 °C in aqueous solution. While, Ibrahim *et al*.^39^ stated that the maximum removal efficiency values of Cd^+2^ ions using marine macroalga *Ulva lactuca* was 84.6 mg/g at pH 5, contact time 60 min, adsorbent dose 0.8 g/L and initial cadmium 60 mg/L. The optimum pH of adsorbing cadmium from water medium using synthetic water and industrial water was at pH 11. Also the optimum pH for adsorption of heavy metals for marine algae also lies between pH 5 - pH 10^54^, where the maximum cadmium removal was recorded at pH 5^39^.

The 3D plot and its corresponding contour plot (Fig. 2B), describes the effects of cadmium concentration (X_1_) and biomass (X_3_) on cadmium removal, while pH (X_2_) was kept at its zero level (7). It is evident from Fig. 2B that the cadmium removal increased at biomass concentration beyond 2.5 g/L after which cadmium removal decreased. Lower and higher levels of cadmium concentration (X_1_) support a relatively low percentage of cadmium removal and the maximum percentage of cadmium removal clearly situated close to the central point of cadmium concentration. By solving the Equation (2) and analysis of Fig. 2B, the maximum predicted cadmium removal of 100.07% was obtained at the optimum predicted levels of cadmium concentration and biomass of 200 mg/L and 2.6; respectively at pH 7.

Percentage of cadmium ions removal increased with increases in adsorbent concentration (biomass). The increase in the removal of metal ions with an increase in the adsorbent concentration is mostly attributed to an increase in the metal sorption surface area and the availability of more adsorption active sites on the surface of the adsorbent^55^. The maximum metal adsorption at a higher adsorbent concentration may be due to the higher active sites for *Ulva lactuca* that enhance the removal of metal ions^41,42^. Lesser adsorption capacity at higher adsorbent concentrations may be attributed to agglomeration and a consequent reduction in intercellular distance resulting in the decrease in total adsorbent surface area and adsorption sites available to metal ions^55,56^.

The 3D plot and its corresponding contour plot (Fig. 2C), highlight the roles played by biomass concentration (X_3_) and pH (X_2_) on cadmium removal, when cadmium concentration (X_1_) was kept at its zero level (200 mg/L). As the value of biomass concentration and pH increased, the cadmium removal increased gradually to the optimum level and thereafter the cadmium removal decreased. By solving the Equation (2) and analysis of Fig. 2C, the maximum predicted cadmium removal of 102.47% was obtained at the optimum predicted levels of pH and biomass of 5 and 3; respectively at cadmium concentration 200 mg/L.

According to second-order polynomial models, optimum conditions for maximum removal of cadmium by *Ulva fasciata* from aqueous solution was found under the condition of initial cadmium concentration of 200 mg/L, temperature 25 °C, pH 5, *Ulva fasciata* biomass of 4 g/L and contact time of 60 min at static condition. Under these conditions, the maximum removal percentage of cadmium was 99.96%.

The optimum conditions for maximum removal of cadmium by *Cystoseria myricaas* from aqueous solutions were observed at cadmium concentration of 150 mg/L, temperature 40 °C, pH = 3, adsorbent dose of 5 g/L with 75 min contact time. Under these conditions, the maximum removal percentage of cadmium was 87.03%^57^. Henriques *et al*.^58^ reported that the effectiveness of living *Ulva lactuca* in metal uptake and the removal of cadmium from the contaminated water were confirmed and the removal efficiency was 57%. Results of the previous study by Henriques *et al*.^59^ evidenced the high potential of living *Fucus vesiculosus* to remove cadmium from contaminated salt waters and it was able to reduce the concentrations of cadmium between 25 and 76%. *Spirulina platensis* dry biomass was used as biosorbent for cadmium ions removal from aqueous solutions. High levels of removal (87.69%) were obtained at pH 8, 26 °C, 2 g of biosorbent and 60 mg/L of cadmium concentration after 90 min of contact^60^. Maximum cadmium removal using *Chlorella vulgaris* immobilized on Kappa-carrageenan after 60 min was 66% and on polyurethane foam (Packed bed) was 57%^61^. The highest cadmium removal in green algae was by *Chlorococcum* sp., T5, *Fischerella* sp., *Chlorella vulgaris* var*. vulgaris* and *Scenedesmus acutus* (94, 94, 91, 89 and 88%; respectively). In blue-green algae, highest cadmium removal was by *Lyngbya heironymusii*, *Gloeocapsa* sp., *Phormidium molle*, *Oscillatoria jasorvensis* and *Nostoc* sp. (97, 96, 95, 94 and 94%; respectively)^62^.

### Verification of the model

The optimum conditions for maximum removal of cadmium from aqueous solution by *Ulva fasciata* biomass were contact time of 60 min, initial cadmium concentration of 200 mg/L, pH 5, temperature 25 °C, *Ulva fasciata* dry biomass of 4 g/L with the static condition. Under these conditions, the experimental maximum removal percentage of cadmium (99.96%) was verified and compared with the predicted value from the polynomial model (100.81%). The verification showed a high degree of accuracy of the model of more than 99.16%, demonstrating the model validation under the levels used.

### FTIR Analysis

**Figure 3:**
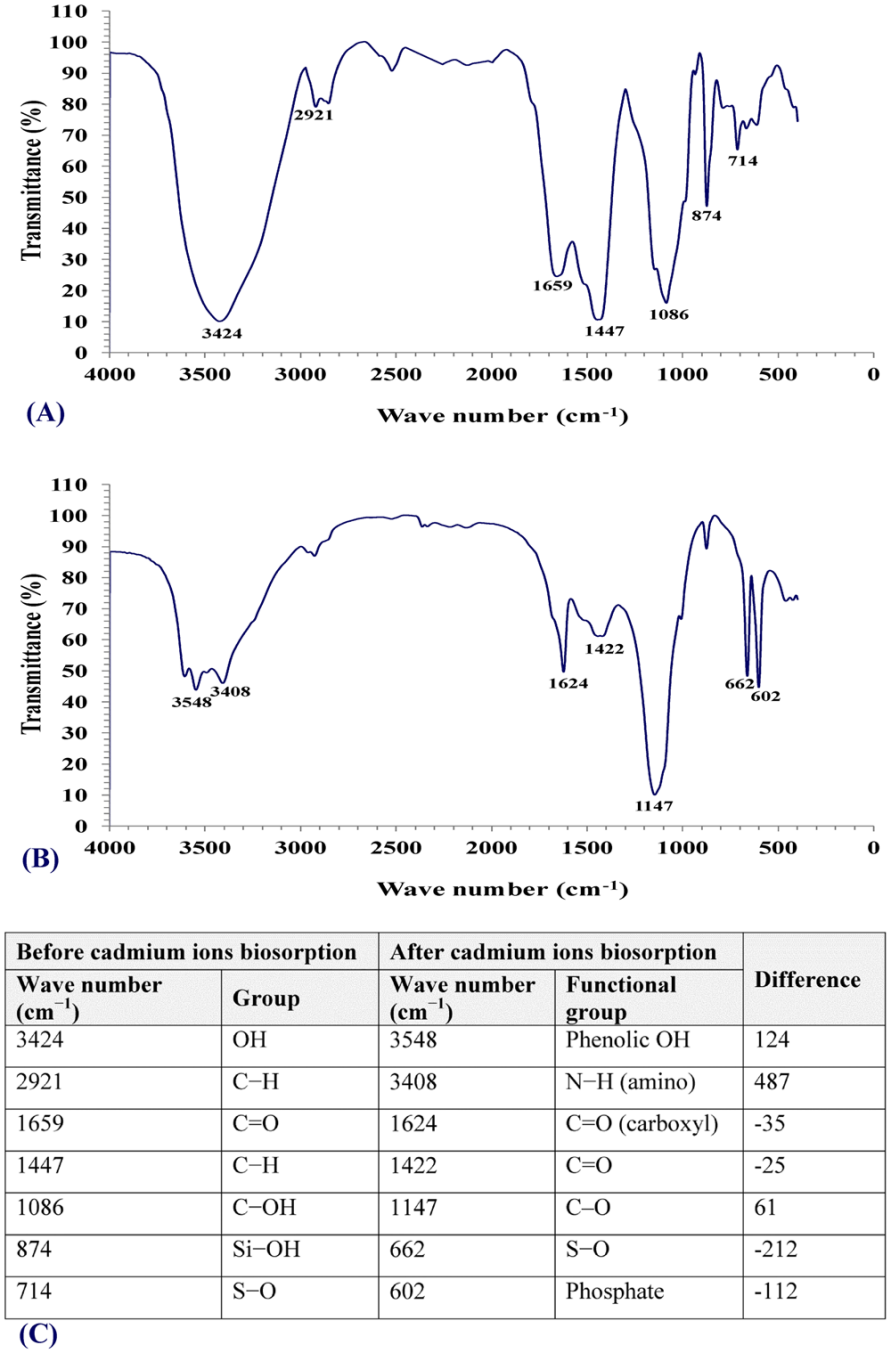
FTIR of *Ulva fasciata* biomass: (**A**) before cadmium ions biosorption; (**B**) after cadmium ions biosorption from aqueous solution; (**C**) summary of wave numbers and corresponding functional groups.

The FTIR spectrums of *Ulva fasciata* dry biomass samples were analyzed before and after cadmium biosorption (Fig. 3) to detect any differences due to the interaction of metal ions with functional groups. The microbial cell walls, which mainly consist of lipids, polysaccharides and proteins, offer many functional groups that can bind heavy metal ions, and these include hydroxyl, carboxylate, phosphate and amino groups^27^. Metal ions biosorption occurs on the surface of the cell by means of an ion exchange method. The spectra of adsorbents were measured within the range of 400–4000 cm^−1^ wave number^40^. FTIR spectrum for dry biomass sample before cadmium biosorption showed the characteristic absorption peaks at 3424, 2921, 1659, 1447, 1086, 874 and 714 cm^−1^. The peak in pure biomass sample before cadmium biosorption at 3424 cm^−1^ is assigned to O–H groups^63^. The peak at 2921 cm^−1^ can be assigned to C–H group belonging to lipids and phospholipids fractions^64^. The band at 1659 cm^−1^ is assigned to C = O stretching^65^. The band at 1447 cm^−1^ related to carbonate carboxylate or methyl groups^66^. The characteristic absorption peak at 1086 cm^−1^ representing C-OH stretching vibrations^67^. The bands at 874, 714 are assigned to Si-OH, S-O; respectively.

FTIR spectrum for biomass sample before cadmium biosorption showed the previously mentioned characteristic absorption peaks at 3424, 2921, 1659, 1447, 1086, 874 and 714 cm^−1^ were shifted to 3548, 3408, 1624, 1422, 1147, 662 and 602 cm^−1^ after cadmium biosorption by the biomass. These results show the association of these functional groups in the biosorption of cadmium ions. The peak at 2921 cm^−1^ representing C-H stretching vibrations shifted to 3408 cm^−1^ which assigned to the stretching vibration of N−H. The carboxylic acid (C = O) band at 1659^−1^ is moved to lower wavelength at 1624 ^−1^, the variation in the wave number reveals, that the involvement of the carboxylic acid in the ion exchange process. The peak at 1447 cm^−1^ representing C-H stretching vibrations shifted to the peak at 1422 cm^−1^ which assigned to the stretching vibration of C = O^68^. Additionally, the peak at 714 cm^−1^ shifted to 602 cm^−1^ could be assigned to the bending modes of alcoholic hydroxyl (–OH)^69^. The band moved from 1086 cm^−1^ representing C-OH stretching vibrations to 1147 cm^−1^ corresponding to stretching vibration of C–O^70^. In conclusion, FITR confirmed that the carboxylic, phenolic, and amide groups were the main groups involved in the cadmium ions biosorption process. These changes in the wave numbers and intensity were as a result of complexation or coordination between cadmium ions and the functional groups on the *Ulva fasciata* biomass.

### Scanning electron microscopy (SEM)

**Figure 4:**
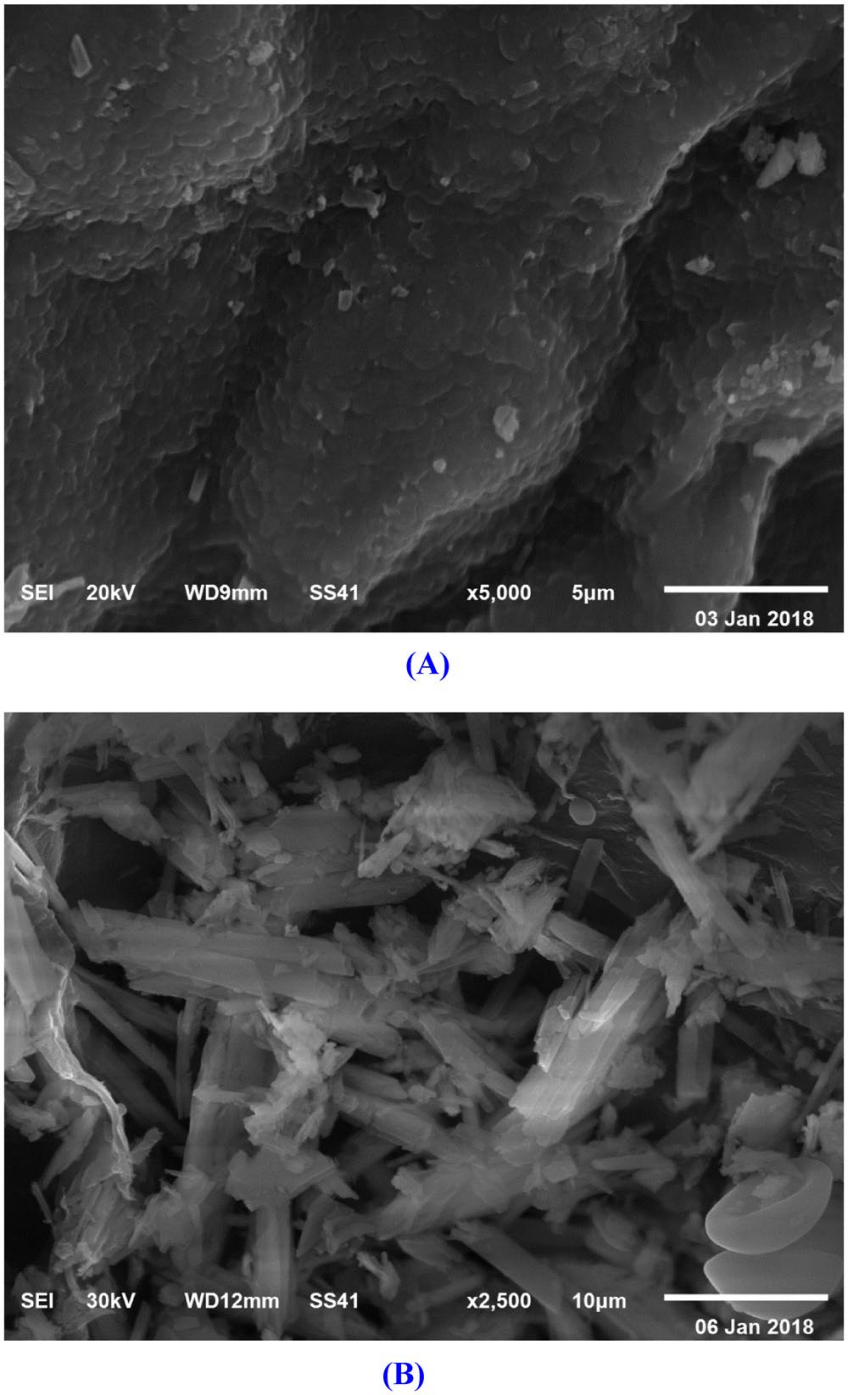
SEM micrograph of *Ulva fasciata* biomass: (**A**) before and (**B**) after adsorption of cadmium ions from aqueous solution.

Figure 4A shows SEM micrograph of *Ulva fasciata* biomass before adsorption of cadmium ions and Fig. 4B shows SEM micrograph of *Ulva fasciata* biomass after adsorption of cadmium ions. The graphs demonstrate the ability of *Ulva fasciata* biomass to adsorb and remove cadmium from aqueous solutions.

### Electron dispersive spectroscopy (EDS)

**Figure 5:**
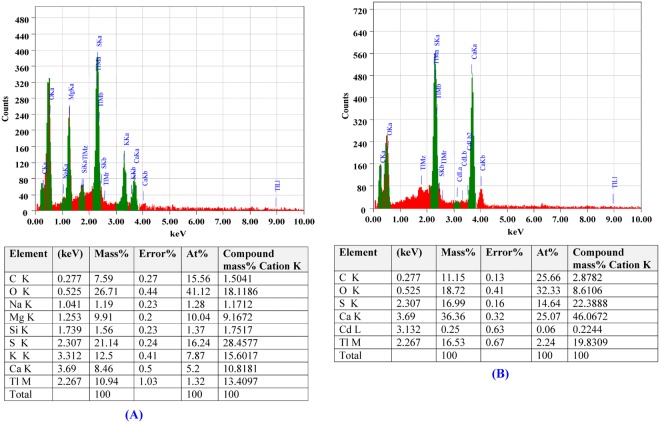
EDS analysis for *Ulva fasciata* biomass (**A**) before cadmium ions biosorption; (**B**) after cadmium ions biosorption from aqueous solution.

Energy-dispersive spectroscopy (EDS) is a useful tool used for the elemental analysis or chemical characterization of biosorbents^71^. In the present study, the EDS analysis was performed to confirm the presence of cadmium attached to the cell surface of *Ulva fasciata* biomass. Figure 5 clearly indicated the presence of additional optical absorption peak corresponding to the cadmium after the biosorption which confirms the ability of *Ulva fasciata* biomass to remove cadmium ions from aqueous solutions. This result is in agreement with the result reported by Huang *et al*.^72^, who observed that cadmium ions accumulated on the surface of dead cells.

### Immobilization of *Ulva fasciata*in alginate beads and its application in cadmium removal

**Figure 6:**
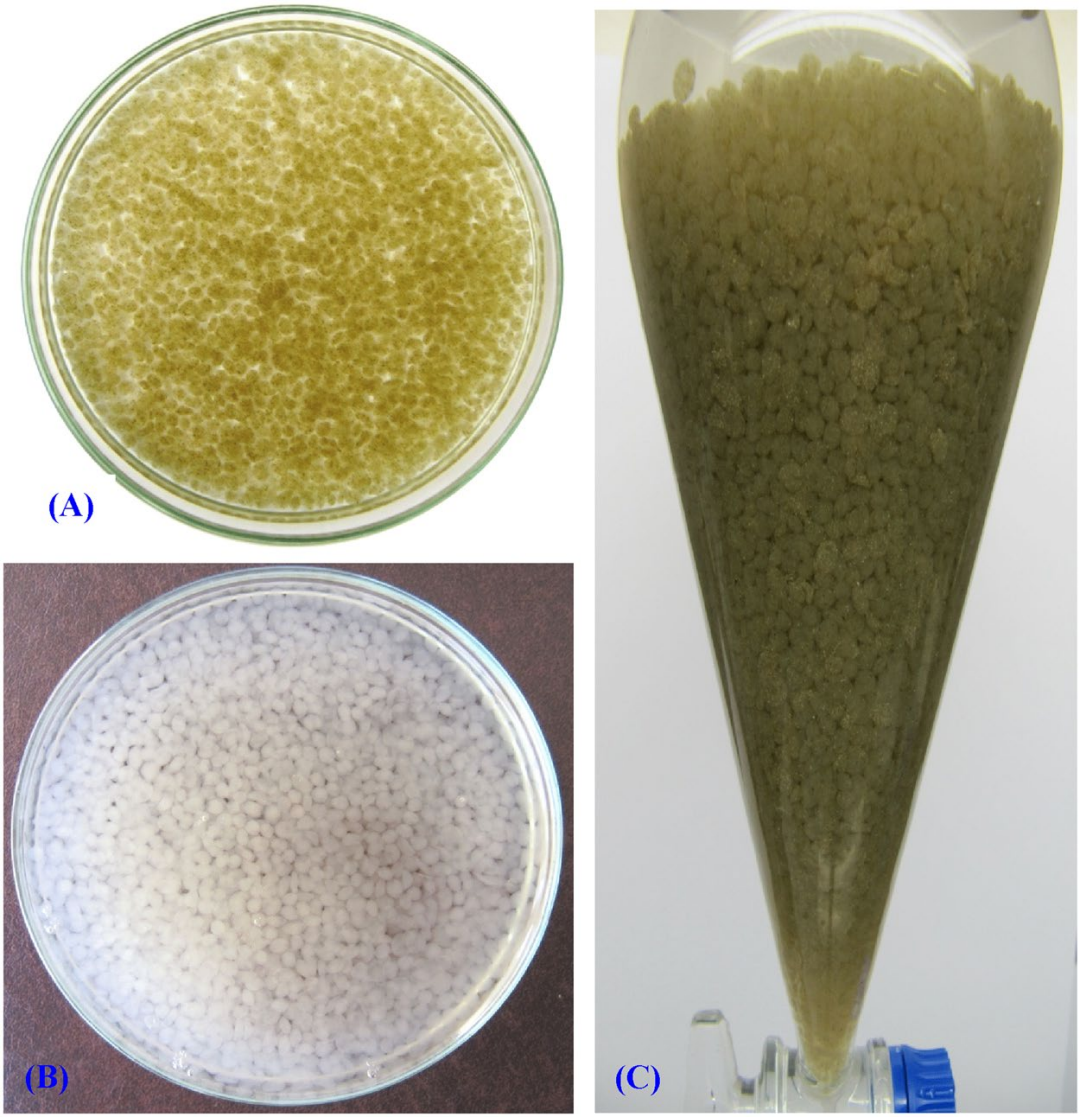
Immobilization of *Ulva fasciata* in alginate beads and its application in cadmium removal from aqueous solution. (**A**) Sodium alginate-*Ulva fasciata* biomass beads; (**B**) Sodium alginate beads without incorporation of the algal biomass; (**C**) Separating funnel packed with alginate- algal beads.

**Figure 7:**
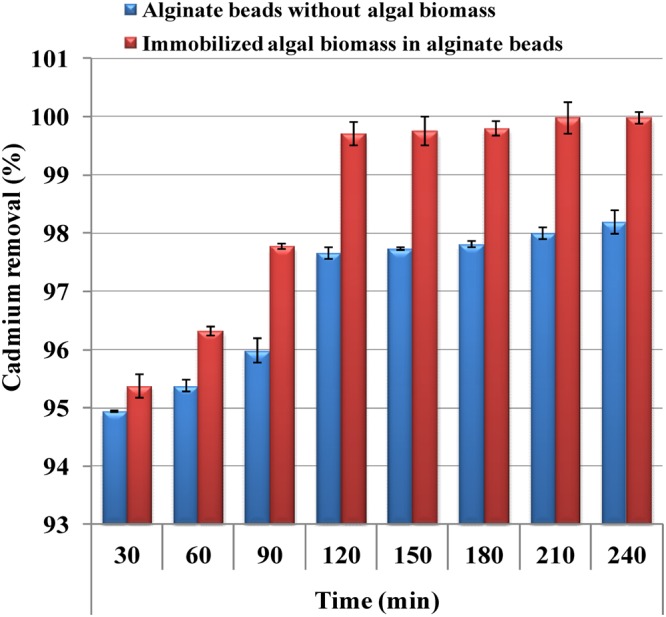
Application of immobilized *Ulva fasciata* biomass in cadmium removal from aqueous solution.

Polysaccharide gel-immobilized algal cells are often used to remove phosphates, nitrates and heavy metal ions from wastewater, providing an alternative to existing physicochemical wastewater treatment technologies^73^. The ability to remove cadmium in aqueous solution by the immobilized *Ulva fasciata* in sodium alginate beads was studied (Fig. 6) and the results were represented in Fig. 7. The results indicated that the treatment of aqueous solution containing cadmium with immobilized *Ulva fasciata* biomass in sodium alginate-beads removed 99.98% of cadmium at an initial concentration of 200 mg/L after 4 h, which is significantly higher than the removal presented using sodium alginate beads without incorporation of the algal biomass as a control (98.19%) (Fig. 7). The ability to remove cadmium in aqueous solution by immobilized *Chlorella* sp. in calcium alginate beads was studied by Valdez *et al*.^74^ and they mentioned that the treatment with immobilized *Chlorella* sp. in alginate beads removed 59.67% of cadmium at an initial concentration of 20 ppm at 80 min, which is significantly higher than the removal presented using beads without *Chlorella* sp. as a control (55.56%). Some studies reported that the use of immobilized cells is more efficient in removing metals than free cells^75–78^. Size of the bead used for biomass immobilization is an important factor^79^. Immobilized biomass of *Sargassum baccularia* in polyvinyl alcohol used for the lead and copper removal has been reported by Tan *et al*.^80^. Tam *et al*.^81^ reported that the bioreactors containing alginate algal beads were more effective in removing N and phosphate from wastewater than blank alginate beads. In another study, the microspheres ensure a removal of 100% of the initial metals quantities at low concentrations (up to 10 mg/L) and the maximum removal capacity at the higher initial concentration (200–300 mg/L) is 70% for lead and 82% for cadmium^82^. Beads can be used in successive biosorption/desorption cycles for the removal of metal ions from the medium, which suggested that immobilization can offer an effective and appropriate technique for repetitive use of biosorbent^83^.

## Material and Methods

### Collection of marine alga and biomass preparation

*Ulva fasciata* was collected from Abukir beach, Alexandria governorate, Egypt. The collected alga was washed with tap water then twice with distilled water for removal of the external sand and salts. The alga was dried in an oven at 70 °C for 72 hrs, and then milled with a blender, sieved to get particle with the size pass through a laboratory test sieve (Endecotts Ltd., London, England) with a mesh size of 125 µm (Supplementary Fig. S1). 20 g of the dried biomass of *Ulva fasciata* were thoroughly mixed with 1 L of distilled water in 2 L Erlenmeyer flask and the suspension was incubated with shaking at 250 rpm at room temperature. At the completion of incubation in shaking condition, the biomass was filtered and dried at 70 °C for 72 hours or until constant weight was obtained and kept at room temperature for further use in biosorption experiments.

### Preparation of heavy metal solution

A defined concentration of cadmium ions (25,100, 200, 300 mg/L) were dissolved in 100 mL of distilled water. The initial pH was adjusted with 0.1 N H_2_SO_4_ and 0.1 N NaOH.

### Statistical optimization for cadmium removal by Plackett-Burman design

Plackett-Burman design was used as a screening method to identify the most significant variables with respect to their main effects^84,85^. The Plackett–Burman statistical experimental design^86^ is a two-factorial design (−1 for low level and +1 for high level), which identifies those factors that had a significant effect, either positively or negatively on cadmium biosorption. The experiment was conducted in 12 runs to study the effect of the selected six variables on the cadmium removal efficiency. The selected six variables were cadmium ions concentration (25 and 200 mg/L), biomass concentration (1 and 4 g/L) at two initial pH levels (4 and 7) which was adjusted with 0.1 N H_2_SO_4_ and 0.1 N NaOH, different contact times (60 and 180 min), static or agitation condition and temperatures 25 and 50 °C were carried out.

Dry biomass was thoroughly mixed with cadmium solution in Erlenmeyer flasks. The suspensions were kept static or with agitation for specific contact time at the selected temperature. The contact time intervals for sampling were 60 or 180 min. After each contact time interval, the residual cadmium concentration in the solution was determined by using inductively coupled plasma – atomic emission spectroscopy (ICP-AES, Thermo Scientific).

Plackett–Burman experimental design is based on the first order model:

3 $$Y={\beta }_{0}+\sum \,{\beta }_{1}{X}_{i}$$

Where, Y is the measured response (cadmium removal percentage), β_0_ is the model intercept and *β*_*i*_ is the linear coefficient, and X_i_ is the level of the independent variables. All experiments were carried out in triplicate.

### Optimization of cadmium removal by response surface methodology

The face centered central composite design (FCCD) was employed to study the interactions effects between the significant variables and to determine their optimal levels^87^. The optimum levels of three significant variables (cadmium concentration, pH and biomass concentration) and to study the individual and mutual interactions among the tested variables on the cadmium removal. The FCCD is a statistical experimental design where each factor is varied on three different levels, low (−1), medium (0), high (+1) and 6 runs at the midpoint, resulting in a total of 20 trials. The second-degree polynomial equation was used to calculate the relationship between the independent variables and the cadmium removal. Given all linear, square and interaction coefficients, the quadratic regression model can be illustrated as follows:

4 $$Y={\beta }_{0}+\sum _{i}\,{\beta }_{1}{X}_{i}+\sum _{ii}\,{\beta }_{ii}{X}_{i}^{2}+\sum _{ij}\,{\beta }_{ij}{X}_{i}{X}_{j}$$

In which Y is the predicted response, β_0_ is the regression coefficients, β_i_ is the linear coefficient, β_ii_ is the quadratic coefficients, β_ij_ is the interaction coefficients, and X_i_ is the coded levels of independent variables.

### Statistical analysis

Minitab and Design Expert version 7 for Windows softwares were used for the experimental designs and statistical analysis. The statistical software package, STATISTICA software (Version 8.0, StatSoft Inc., Tulsa, USA) was used to plot the three-dimensional surface plots.

### Analytical methods

10 mL of filtrate from each trial of Plackett-Burman and FCCD were taken, filtered by double rings filter paper (Qualitative medium speed 102, diameter 12.5 cm) and analyzed using inductively coupled plasma – atomic emission spectroscopy (ICP-AES, Thermo Scientific).

The efficiency of alga biomass for cadmium ions removal from aqueous solutions was quantitatively calculated as follows:

5 $${\rm{Removal}}\,{\rm{efficiency}}\,( \% )={{\rm{C}}}_{{\rm{i}}}-{{\rm{C}}}_{{\rm{f}}}/{{\rm{C}}}_{{\rm{i}}}\times 100$$

Where: C_i_ is the initial metal ion concentration (mg/L), C_f_ is the final (residual) metal ion concentration (mg/L). All Determinations of cadmium ions in the solution were carried out in triplicates.

### Fourier transform infrared (FTIR) spectroscopy

The *Ulva fasciata* dry biomass samples were analyzed before and after cadmium biosorption using FTIR to confirm the presence of functional groups in samples. The biomass samples were incorporated with KBr Pellets. The FTIR spectra were measured using Thermo Fisher Nicolete IS10, USA spectrophotometer within the range of 400–4000 cm^−1^.

### Scanning electron microscopy (SEM)

Dry *Ulva fasciata* biomass samples were analyzed before and after cadmium biosorption using SEM to examine the biomass surface and to evaluate the cadmium adsorption. The samples were coated with gold and were examined at different magnifications. The voltage used was 10–30 keV.

### Electron dispersive spectroscopy (EDS)

Energy dispersive spectroscopy analysis (EDS) was carried out with the scanning electron microscope (Oxford X-Max 20) with secondary electron detectors at an operating voltage of 20 kV at Electron Microscope Unit, City of Scientific Research and Technological Applications, Alexandria, Egypt.

### Immobilization of *Ulva fasciata* in alginate beads and its application in cadmium removal

A solution of 4% sodium alginate was prepared by dissolving 4 g sodium alginate into 100 mL distilled water with continuous stirring for 30 min at 60 °C for better dissolution^88^. After cooling, dried and washed 4 g of *Ulva fasciata* biomass sieved by laboratory test sieve (125 µm, Endecotts Ltd., London, England) was added with stirring at room temperature for 5 min. The beads of 1.5 ± 0.2 mm diameter were obtained by adding drop-wise of the alginate algal biomass mixture through 3 mL syringe into a cold sterile 2.5% calcium chloride solution in distilled water under gentle stirring at room temperature. The resulting beads were washed several times with autoclaved distilled water to remove any remains of calcium chloride from the beads surfaces and later stored overnight at 4 °C in distilled water in order to stabilize and harden the beads. In the same way, sodium alginate beads without incorporation of the *Ulva fasciata* biomass are also prepared and used as the control to determine the adsorption capacity of the alginate algal beads for cadmium ions. For storage, the beads were dipped in 0.2 M of Tris-HCl buffer (pH 7.2) and stored at 4 °C until further use.

Separating funnel experiment was conducted in 250 mL Simax glass separating funnel packed with alginate algal beads. Cadmium solution (200 mg/L) was added to the separating funnel. Samples (5 mL) from the separating funnel effluent were collected regularly (every 30 min for up to 4 hours) at a flow rate of 3 mL/min and analyzed by inductively coupled plasma – atomic emission spectroscopy (ICP-AES, Thermo Scientific). The biosorption capacity of the cadmium ions was determined by the difference in cadmium solution concentration before and after adsorption.

## Conclusion

Cadmium is a toxic industrial and environmental pollutant heavy metal classified as a human carcinogen. The biosorption conditions of cadmium ions from aqueous solutions using *Ulva fasciata* dry biomass as adsorbent were optimized by using Plackett–Burman and face centered central composite designs. Optimum levels of the factors obtained for maximum cadmium removal (99.96%) were contact time of 60 min, initial cadmium concentration of 200 mg/L, pH 5, temperature 25 °C, *Ulva fasciata* dry biomass of 4 g/L with static condition. FITR confirmed that the carboxylic, phenolic, and amide groups were the main groups involved in the cadmium ions biosorption process. SEM micrograph of *Ulva fasciata* biomass after adsorption of cadmium ions demonstrates the ability of *Ulva fasciata* biomass to adsorb and remove cadmium from aqueous solutions. EDS analysis confirms the ability of *Ulva fasciata* biomass to remove cadmium ions from aqueous solutions. The treatment of aqueous solution containing cadmium with immobilized *Ulva fasciata* biomass in sodium alginate-beads removed 99.98% of cadmium at an initial concentration of 200 mg/L after 4 h. Dry biomass of *Ulva fasciata* could be used as an efficient and cheap biosorbent for cadmium ions removal from polluted water and the process used is safe, feasible and eco-friendly.

## Electronic supplementary material

Supplementary materials
