# Supplementary material for: Statistical optimization for cadmium removal using Ulva fasciata biomass: Characterization, immobilization and application for almost-complete cadmium removal from aqueous solutions
Source: Sci Rep. 2018 Aug 20;8:12456. doi: 10.1038/s41598-018-30855-2 (PMC6102212; doi:10.1038/s41598-018-30855-2)
Supplement: Supplementary file 1 — Supplementary materials [file 41598_2018_30855_MOESM1_ESM.docx]

**Statistical optimization for cadmium removal using *Ulva fasciata* biomass: Characterization, immobilization and application for almost-complete cadmium removal from aqueous solutions**

**^1^Noura El-Ahmady El-Naggar, ^2^Ragaa A. Hamouda,** **^3^Ibrahim E. Mousa, ^2^Marwa Salah Abdel-Hamid and ^2^Nashwa H. Rabei**

^1^Department of Bioprocess Development, Genetic Engineering and Biotechnology Research Institute, City of Scientific Research and Technological Applications, Alexandria, Egypt.

^2^Microbial Biotechnology Department, Genetic Engineering and Biotechnology

Research Institute, University of Sadat City, Egypt

^3^Environmental Biotechnology Department, Genetic Engineering and Biotechnology Research Institute (GEBRI), University of Sadat City, 22857, Egypt.

*To whom correspondence should be addressed.

**Dr. Noura El-Ahmady Ali El-Naggar**

**Address:**

Bioprocess Development Department,

Genetic Engineering and Biotechnology Research Institute,

City of Scientific Research and Technological Applications,

New Borg El- Arab City, 21934, Alexandria, Egypt

**Tel:** (002)01003738444

**Fax:** (002)03 4593423

**E-mail:** [nouraelahmady@yahoo.com](mailto:nouraelahmady@yahoo.com)

**Supplementary Table S1. Fit summary for experimental data**

| **Sequential Model Sum of Squares** | | | | | | | |
| --- | --- | --- | --- | --- | --- | --- | --- |
| **Source** | | **Sum of Squares** | ***df*** | **Mean Square** | | ***F-*value** | ***P-*value**  ***P*rob >*F*** |
| Linear vs Mean | | 112.318 | 3 | 37.439 | | 2.455 | 0.1006 |
| Two factors interaction (2FI) vs Linear | | 123.807 | 3 | 41.269 | | 4.463 | 0.0230* |
| Quadratic vs 2FI | | 107.265 | 3 | 35.755 | | 27.642 | < 0.0001* |
| Residual | | 12.229 | 6 | 2.038 | |  |  |
| **Lack of Fit Tests** | | | | | | | |
| **Source** | **Sum of Squares** | | ***df*** | **Mean Square** | | ***F-*value** | ***P-*value**  ***P*rob >*F*** |
| Linear | 238.233 | | 11 | 21.658 | | 18.757 | 0.0023* |
| Two factors interaction (2FI) | 114.426 | | 8 | 14.303 | | 12.388 | 0.0066* |
| Quadratic | 7.162 | | 5 | 1.432 | | 1.241 | 0.4094 |
| Pure Error | 5.773 | | 5 | 1.155 | |  |  |
| **Model Summary Statistics** | | | | | | | |
| **Source** | **Standard deviation** | | **R-Squared** | | **Adjusted R-Squared** | **Predicted R-Squared** | **PRESS** |
| Linear | 3.9052 | | 0.3152 | | 0.1868 | -0.3150 | 468.5745 |
| Two factors interaction (2FI) | 3.0407 | | 0.6627 | | 0.5070 | -0.1092 | 395.2302 |
| Quadratic | 1.1373 | | 0.9637 | | 0.9310 | 0.8776 | 43.6284 |
| * Significant values,  *df* : degree of freedom, PRESS: sum of squares of prediction error | | | | | | | |

**
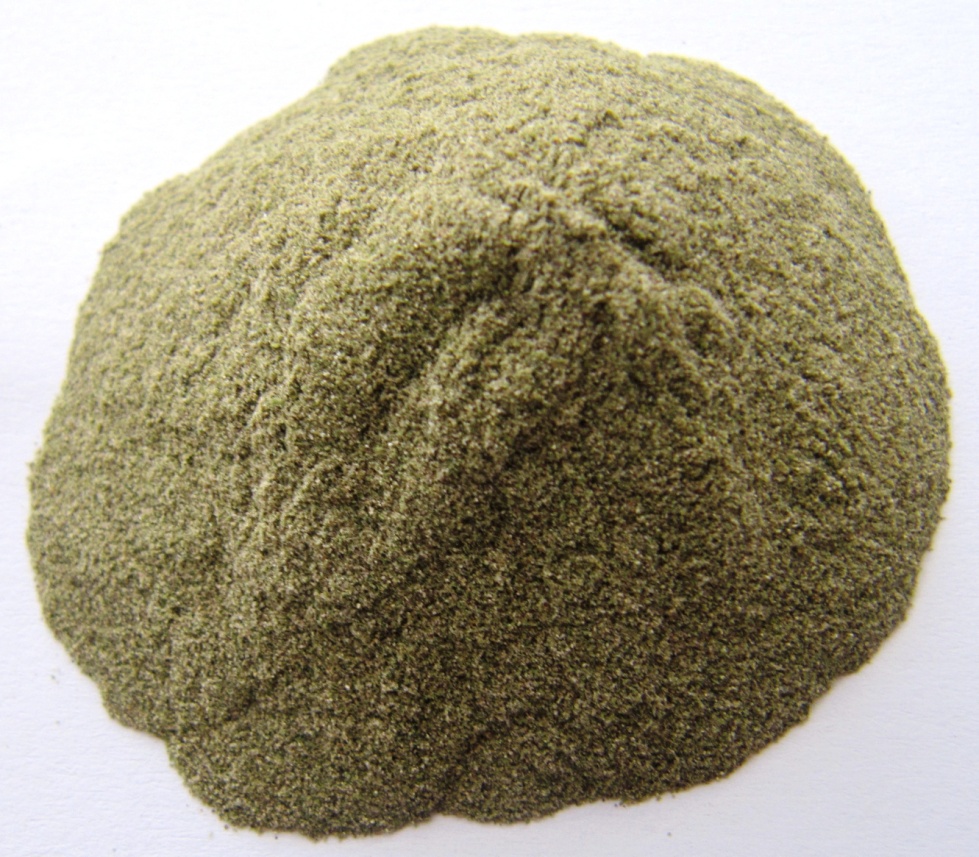
**

**Supplementary Figure S1.** The dried, milled biomass of *Ulva fasciata* with size of 125 µm.

**Supplementary Figure S2.** Estimated effects of independent variables on cadmium removal by *Ulva fasciata* using Plackett-Burman design “the red color represents the most significant positive independent variables affecting cadmium removal”.
